# FAAH inhibition ameliorates breast cancer in a murine model

**DOI:** 10.18632/oncotarget.28534

**Published:** 2023-10-31

**Authors:** Mallika Tripathy, Amy Bui, Jared Henderson, Jeffrey Sun, Christian Rutan Woods, Soumya Somani, Thao Doan, Anto Sam Crosslee Louis Sam Titus, Chandra Mohan

**Affiliations:** ^1^Department Biomedical Engineering, University of Houston, Houston, TX 77204, USA

**Keywords:** FAAH, breast cancer, cancer therapy, apoptosis

## Abstract

Breast cancer is the leading cancer among females worldwide. Disease outcome depends on the hormonal status of the cancer and whether or not it is metastatic, but there is a need for more efficacious therapeutic strategies where first line treatment fails. In this study, Fatty Acid Amide Hydrolase (FAAH) inhibition and endocannabinoids were examined as therapeutic alternatives. FAAH is an integral membrane enzyme that hydrolyzes endocannabinoids, rendering them inactive, and FAAH inhibition is predicted to increase cancer cell death. To test this, breast cancer cells were probed for FAAH expression using Western blot analysis, treated with FAAH inhibitors, exogenous endocannabinoids, and combinations of the two treatments, and assessed for viability. High levels of FAAH were observed in different breast cancer cell lines. FAAH inhibition was more effective than exogenous endocannabinoid treatment, and the combination of FAAH inhibitors and endocannabinoids was the most effective in inducing apoptosis of breast cancer cells *in vitro*. In addition, *in vivo* FAAH inhibition reduced breast cancer growth in immunodeficient mice. FAAH inhibition is a promising approach, and tremendous progress has been made in the field to validate this mechanism as an alternative to chemotherapy. Further research exploring the therapeutic potential and impact of FAAH expression on cancer cells is warranted.

## INTRODUCTION

Our previous studies have implicated fatty acid amide hydrolase (FAAH) as a disease gene for autoimmunity, where heightened FAAH expression drives B-cell survival and B-cell driven autoimmunity. In relation to autoimmunity, FAAH was also found to be upregulated in B-cells and led to a reduction in the number of polyreactive autoantibodies in lupus-prone mice [1]. Given this lead, we proceeded to examine if FAAH might also play a role in malignancies. FAAH is an integral membrane protein that functions in hydrolyzing fatty acid amides, such as endocannabinoids. Due to its function in the endocannabinoid pathway, FAAH has been studied in several different contexts. Inhibitors of the enzyme have led to analgesia, anti-inflammatory, and antidepressant effects [2]. Among cancer patients, the activity of FAAH was also reported to be upregulated [3]. Moreover, FAAH inhibition has been analyzed in lung cancer [4], prostate cancer [5], and colorectal cancer [6], and found to be beneficial.

Endocannabinoids are part of a biological system that exists throughout the body. It serves as a regulatory system to ensure homeostasis is maintained, and works to regulate temperature, blood sugar levels, pH, as well as water, mineral, and metabolic waste balance. These are lipid-based atypical neurotransmitters that are synthesized based on specific interactions between the neurotransmitter and receptor. Once they are released into the extracellular space, they can be taken up into cells and degraded by FAAH [7]. The two major endocannabinoids currently studied are anandamide (AEA) and 2-arachidonoylglycerol (2-AG), which both bind with high affinity to cannabinoid (CB) CB1 and CB2 receptors [8]. Indeed, 2-AG and AEA have been reported to inhibit human breast cancer cell proliferation [9]. AEA has also been shown to inhibit human breast cancer cell proliferation *in vitro* through CB1-like receptor-mediated inhibition. While the role of FAAH in endocannabinoid-mediated nociception for breast cancer has been studied, its role in cancer growth and progression has not been examined.

**Figure 1:**
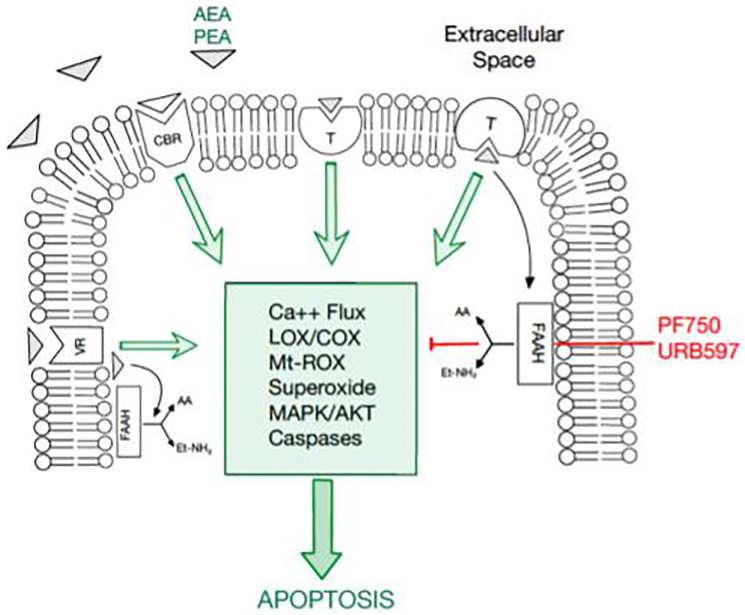
An overview of the endocannabinoid pathway and molecules tested in this study. The cellular apoptosis cascade is mediated by a multitude of molecules and receptor-ligand interactions. Fatty acid amide hydrolase (FAAH) has been documented to inhibit the apoptosis pathway. Thus, FAAH inhibitors, PF750 and URB597, have promising potential in inducing apoptosis in breast cancer cells. Moreover, cannabinoids, such as AEA and PEA, promote apoptosis, serving as another potential therapeutic agent in cancer cells.

Since FAAH in cancer cells modulates the apoptotic potential of endocannabinoids by promoting endocannabinoid hydrolysis, it was hypothesized that high levels of FAAH may be implicated in cancer cell proliferation. FAAH inhibitors promote the endocannabinoid levels of AEA and other fatty acid amides that reduce cancer cell proliferation. Notably, FAAH inhibitors may enhance endocannabinoid tone in certain cells and tissues that release endocannabinoids and are undergoing active synthesis [2]. These factors are known to impact the apoptotic cascade as depicted in Figure 1. Given these observations, treatment with FAAH inhibitors and exogenous cannabinoids have the potential to downregulate cancer cell proliferation. This study explores the functional relevance and therapeutic potential of FAAH inhibition combined with the proapoptotic activity of exogenous endocannabinoids on breast cancer survival.

## RESULTS

### Faah expression in breast cancer

**Figure 2:**
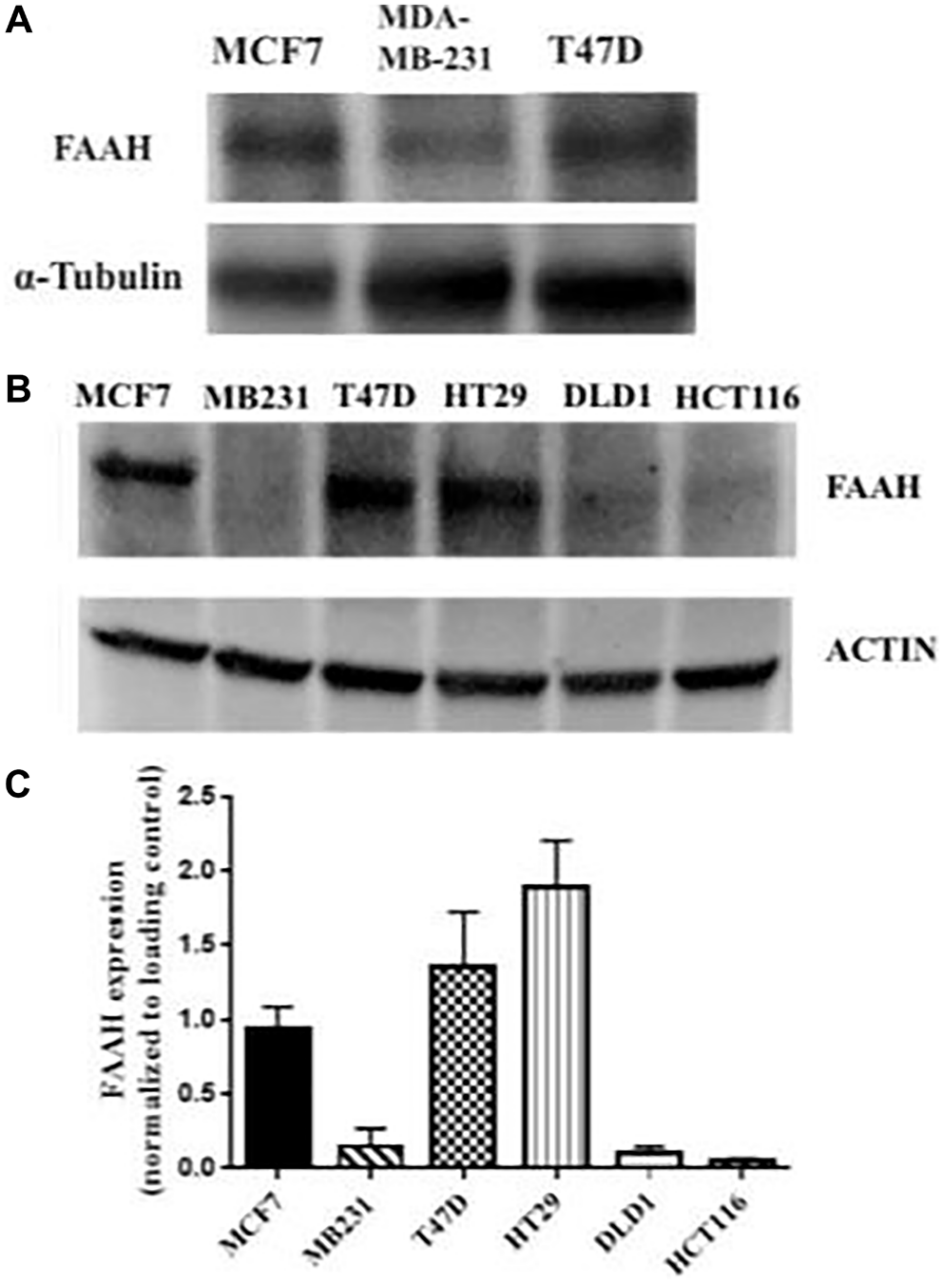
FAAH expression profiles in breast cancer and control cell lines. (**A**) Western blot was conducted using three breast cancer cell lines (MCF7, MDA-MB-231, and T47D) with α-tubulin as the loading control. (**B**) An independent Western blot was conducted using six cell lines. Three of these were breast cancer cell lines (MCF7, MDA-MB-231, and T47D), and the remaining were colorectal adenocarcinoma cell lines (HT29, DLD1, and HCT116). Expression of FAAH in each of the cell lines was measured. Actin was used as the loading control. (**C**) The mean expression of FAAH in the respective cell lines is shown (*N* = 3–6 repeat experiments each).

Three breast cancer cell lines and three colorectal adenocarcinoma cell lines were probed for FAAH expression using Western blot as shown in Figure 2. Western blot analysis confirmed the presence of elevated FAAH in the T47D and MCF7 breast cancer cell lines, especially in the T47D cell line. The MDA-MB-231 breast cancer cell line did not show a similarly elevated expression of FAAH. The HT29 cell line showed high levels of FAAH expression, but the two other colorectal adenocarcinoma cell lines (DLD1 and HCT116) did not. Two independent Western blot studies showed similar findings with high levels of FAAH expression in MCF7 and T47D cell lines and significantly lower levels in the MDA-MB-231 cell line (Figure 2).

Increased expression of FAAH by the breast cancer cell lines may allow for evasion of the apoptosis cascade. If FAAH does confer a survival advantage to cells, then FAAH inhibition would promote apoptosis, especially in those cells that highly express FAAH. Because endogenous cannabinoids promote cellular death, thereby inhibiting unchecked cellular proliferation, it was also hypothesized that adding exogenous cannabinoids would further enhance tumor apoptosis. To test these hypotheses, the three breast cancer cell lines were treated with one of the following treatments: FAAH inhibitors, exogenous cannabinoids, or a combination of an FAAH inhibitor and an exogenous cannabinoid.

### The role of faah inhibition and endocannabinoids in apoptosis

**Figure 3:**
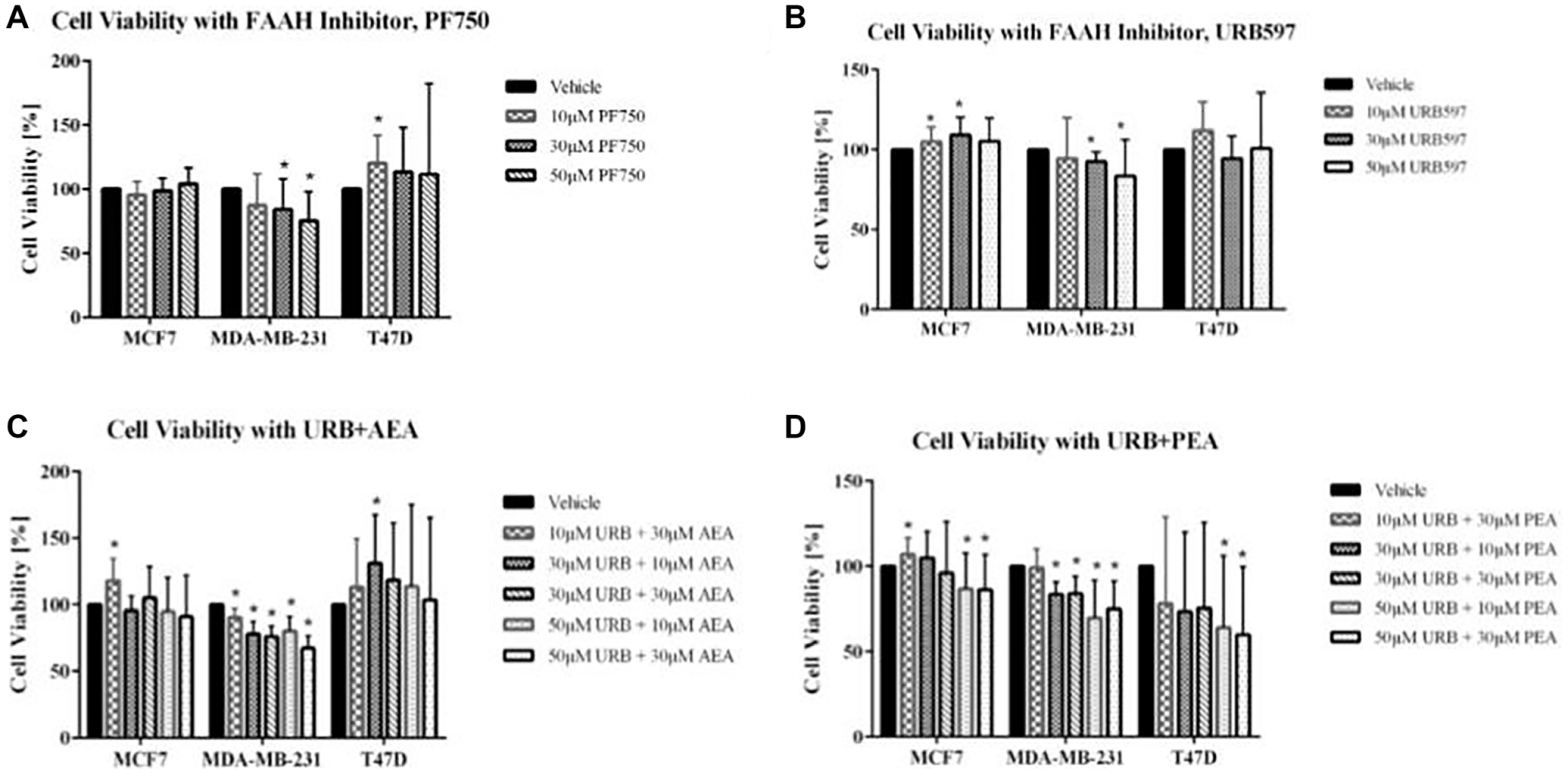
Impact of FAAH inhibitors and exogenous cannabinoids on breast cancer cell viability. (**A**, **B**) Cell viability assays were conducted using the FAAH inhibitors, PF750 and URB597, with the following treatments: vehicle control (cells with media and DMSO), 10 μM concentration, 30 μM concentration, and 50 μM concentration. (**C**, **D**) Cell viability was also measured for combination treatment of FAAH inhibitors with exogenous cannabinoids (URB597 with AEA and URB597 with PEA). For the combination treatments, assays included a vehicle control (cells with media and DMSO), 10 μM URB597 and 30 μM AEA/PEA, 30 μM URB597 and 10 μM AEA/PEA, 30 μM URB597 and 30 μM AEA/PEA, 50 μM URB597 and 10 μM AEA, and 50 μM URB597 and 30 μM AEA. Statistical significance was determined using a non-parametric Mann-Whitney *U*-test (^*^ρ < 0.05; *N* = 3–6 each).

The two FAAH inhibitors tested were PF750 and URB597. As shown in Figure 3A, 3B, decreases in cell viability were not observed for the T47D and MCF7 cell lines, upon addition of these inhibitors. The MDA-MB-231 cell line displayed significant decreases in cell viability after treatment with PF750. In fact, there was a continuous decrease in cell viability with each increase in the PF750 treatment dosage. With URB597, the other FAAH inhibitor used, decreases in cell viability were similarly visualized in the MDA-MB-231 breast cancer cell line (Figure 3C, 3D). This pattern of decreasing cell viability was not seen in the MCF7 and T47D cell lines.

Another treatment option that was evaluated in this study was the utility of exogenous cannabinoid administration. Of relevance, endocannabinoids like AEA have been shown to demonstrate antiproliferative activity in breast cancer cells [10]. The purpose of adding additional cannabinoids exogenously was to increase the quantity of cannabinoids present to promote their anti-inflammatory and anti-survival effects to the maximum. However, possibly because FAAH molecules were still being produced by the cells, exogenous cannabinoids were hypothesized to have minimal effect in reducing cell viability compared to FAAH inhibitors and combination treatments with cannabinoids.

As shown in Supplementary Figure 1, both AEA and PEA, the two cannabinoids tested, had only modest effects on cell viability. For the combination treatments, both exogenous cannabinoids, AEA and PEA, were combined with the FAAH inhibitor URB597 to assess their combined therapeutic benefit. Along with a vehicle control, treatments of 10 μM, 30 μM, and 50 μM URB597 were combined with either 10 μM of AEA or 30 μM of AEA. In the URB597 and AEA combination treatment for MDA-MB-231, considerable decreases in cell viability were noticed. There were incremental decreases in cell viability with increasing dosage of treatment, with the lowest percent viability of the MDA-MB-231 cells being seen with the combination treatment involving the highest doses of both the FAAH inhibitor and exogenous cannabinoids (50 μM URB597 and 30 μM AEA). The other breast cancer cell lines also showed decreases in cell viability with the combination treatment consisting of 50 μM URB597 and 30 μM AEA, but these results were not statistically significant. For the URB597 and PEA combination treatments, all three breast cancer cell lines showed significant therapeutic effects that were expected with combination treatments. In MCF7, MDA-MB-231, and T47D cell lines, there was decreased cell viability at both 50 μM URB597 plus 10 μM PEA and 50 μM URB597 plus 30 μM PEA.

### Faah inhibition effects on tumor growth *in vivo*

While the *in vitro* studies demonstrated FAAH inhibition-driven apoptosis, it is important to translate these findings to an *in vivo* model to further validate the preclinical relevance of the results. Isolated breast cancer cells may rely on other tissues to synthesize most cannabinoids contributing to the endocannabinoid pathway. However, studies have shown that the physiological levels of endocannabinoids reach levels 15 times greater than normal when inhibiting FAAH [11]. Hence, an *in vivo* model of FAAH inhibition could potentially be more physiological.

An immune-deficient mouse model that lacks immune rejection was used to ensure the breast cancer xenograft implantation would be successful [12]. Immune-deficient mice were chosen with a Foxn1^nu^ mutation, which results in defective thymic epithelium development. The Foxn1^nu^ mutant murine model has been shown to accept breast cancer grafts successfully in other studies [13]. 50% Matrigel, 50% PBS was used as an injection substrate to act as a bio-active scaffold, as reported previously.

**Figure 4:**
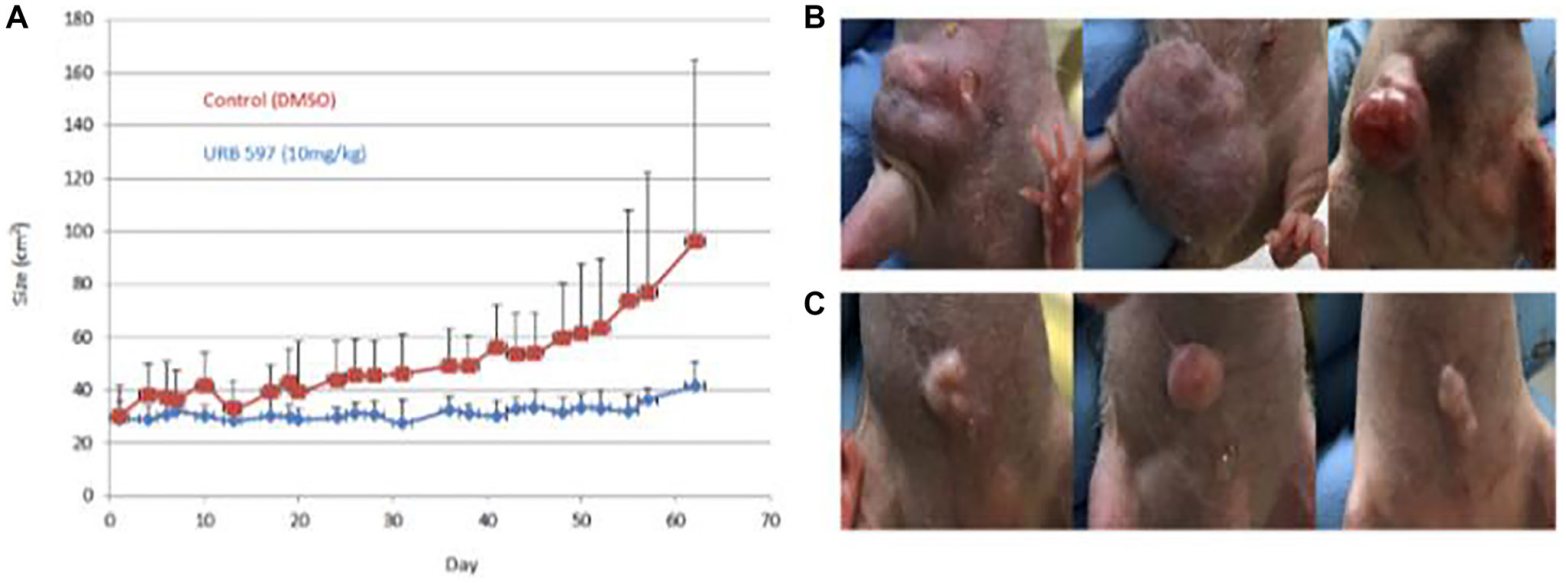
*In vivo* treatment of breast cancer using FAAH inhibitors. Twenty 6-week-old female immune-deficient NU/J mice were aged to 8 weeks before being implanted with 2 million MDA-MB-231 breast cancer cells each. Mice were treated with a vehicle control or the FAAH inhibitor, URB597. Externally visible tumor size was measured over 60 days using calipers and images were also taken at specific timepoints; these are plotted in (**A**). (**B**) Three examples of mice from the vehicle group are displayed. (**C**) Three examples of mice from the drug treatment group are displayed.

Visual comparisons of tumors obtained from mice that were vehicle treated and URB597 treated are presented in Figure 4, as ascertained by caliper measurements were used to monitor the growth of the tumors. Treatment of mice with 10 mg/kg URB597 resulted in a clear decrease in tumor size with the difference in tumor size becoming more prominent with increasing time of treatment, as depicted in Figure 4A. Examples of the tumors are illustrated in Figure 4B, 4C. Although visual inspection and caliper measurements showed a clear difference between the vehicle and FAAH inhibited mice, these differences did not attain statistical significance (Figure 4). Although IVIS imaging was also attempted, technical challenges prohibited the successful completion of these studies.

## DISCUSSION AND CONCLUSION

Besides being the most common, breast cancer is also a leading cause of cancer death in women worldwide [13–18]. The projected incidence and number of deaths due to breast cancer in the US in 2023 are 300,00 and 43,000, respectively [19]. By 2030, the worldwide number of new breast cancer cases will reach 2.7 million annually, with these increases projected to be significantly higher in low- and medium-income countries [19–20]. Treatment of breast cancer and outcome depend on the presence of estrogen or progesterone receptors and human epidermal growth factor 2 (ERBB2) in the tumor [21–25]. 70% of patients have hormone receptor positive/ERBB2 negative tumors, ~15% have ERBB2 positive tumors, while the remaining ~15% have triple-negative tumors. Depending on their molecular type, a combination of endocrine, ERBB2-targeted and chemotherapy is commonly used for treatment of breast cancer, as detailed elsewhere [21–25]. Non-metastatic triple-negative breast cancer is more likely to recur, with a 5-year survival rate of 85%. On the other hand, metastatic triple negative breast cancer has the poorest outcome, with a median overall survival rate of 1 year, compared to 5 years for the other 2 subtypes [21–25]. Interestingly, in parts of the world with advanced health care, the 5-year breast cancer survival is 89.6% for localized and 75.4% for regional disease. In less developed countries, the corresponding survival rates are significantly lower, 76.3% and 47.4%, respectively, taking all breast cancers together [26]. Thus, there is clearly a need to identify novel treatment modalities to treat breast cancer, particularly metastatic and triple negative breast cancer.

In this context, FAAH inhibition and exogenous cannabinoid administration emerge as promising therapeutic alternatives not only because of the mechanisms by which they act, but also because of their side effect profile. Both treatments potentiate natural mechanisms associated with cancer cells to downregulate cellular proliferation. Because FAAH inhibitors prevent FAAH expression, inhibition of endocannabinoid breakdown would be expected to follow. This treatment, at the highest dose, was hypothesized to produce greater decreases in breast cancer cell viability than sole exogenous cannabinoid treatment, as exogenous cannabinoids would only be increasing endocannabinoid levels without having any effect on the endocannabinoid breakdown that is mediated by FAAH. Even more than FAAH inhibitor treatment, though, the most beneficial effects were expected to occur with combination treatments at the highest concentrations of both the FAAH inhibitor and the exogenous cannabinoid. This treatment would not only prevent the breakdown of the existing endocannabinoids produced by cancer cells but would also add more cannabinoids – two mechanisms by which endocannabinoid concentration would be increased and apoptosis would be promoted.

The cell viability assays presented in this report indicate that the greatest efficacy was observed when the highest concentrations of the FAAH inhibitor, URB597, and exogenous cannabinoids, AEA and PEA, were used. Specifically, the treatment that was most beneficial was that using 50 μM URB597 and 30 μM PEA/AEA. Because of the two mechanisms involved in increasing the endocannabinoid concentrations, these results were expected by the combination treatment. In addition to treating the cells with both therapeutic options, decreases in cellular viability were also noted with both FAAH inhibitor treatments, most notably with the highly selective PF750 FAAH inhibitor. Consistent with our hypothesis and the *in vitro* data, *in vivo* treated mice had evidently smaller tumors, although these differences did not attain statistical significance. The promising trend, however, calls for the repetition of these studies with larger sample sizes, higher drug dose, and bioluminescence-based serial tumor tracking *in vivo*. No adverse side effects were noted from the treatment, suggesting its safety as a therapeutic option.

There has been significant development in understanding the mechanism of action of cannabinoid agonists, such as AEA and its metabolic-stable analogous in various cancers, including breast cancer [27–35]. Mechanisms that may be at play in reducing tumor growth include reduction of β-catenin nuclear translocation and transcriptional activity, downregulation of β-catenin target genes, reduction of mesenchymal transition, modulation of Wnt signaling, activation of Fas-dependent and independent apoptosis pathways, cell cycle arrest, DNA damage, activation of p53 signaling, and modulation of RhoA and MAPK signaling [27–35]. MDA-MB-231 breast cancer cells, used for study in this report, model metastatic estrogen receptor-negative breast cancer. Indeed, in these same breast cancer cells, cannabinoid agonists have been shown to inhibit tumor proliferation by inducing S phase cell cycle arrest, DNA damage and Chk1 activation, and inhibit tumor migration by modulating FAK/SRC/RhoA signaling [33–34]. Others have reported that cannabinoids can disrupted HER2-CB2R complexes by selectively binding to CB2R, thus reducing breast cancer growth [34]. Cannabinoid agonists may also interact with the cannabinoid receptor CB1 to inhibit growth of triple negative breast cancer cells by activating p53 signaling [35]. Given these observations, we hypothesize that FAAH inhibition and cannabinoid agonists may be particularly efficacious in patients with metastatic triple negative breast cancer.

There are limitations associated with this study. With the *in vivo* experiment, the initial plan entailed completing serial IVIS bioluminescence imaging due to its noninvasive method of tracking tumor size without sacrificing the mouse; however, the imaging did not work due to technical hurdles. Serial non-invasive imaging of the tumor would have been another way to track tumor size in addition to the direct measurements. Another limitation with the *in vivo* treatments was that only URB597 was administered, at a single dose — it would have been useful to use PF750 as another FAAH inhibitor being assessed, as well as to conduct experiments with exogenous cannabinoid treatments as was done with the *in vitro* experiments. Understanding the level of FAAH expression in normal tissue would also be important in determining if FAAH expression is higher in cancer cell lines, as well as to understand the implications of inhibiting FAAH. For example, if FAAH is highly expressed in normal breast tissue, then one would need to be mindful of the effects of inhibiting FAAH on normal tissue. Finally, studies are also warranted to examine FAAH expression in primary breast cancer tissue.

## MATERIALS AND METHODS

### Cell lines

The human breast cancer cell lines used in the viability assays were MCF7, MDA-MB-231, and T-47D, which were donated by Dr. Chin-Yo Lin from the Center for Nuclear Receptors and Cell Signaling in the Biology and Biochemistry Department at the University of Houston. Three additional control cell lines, HT29, DLD1, and HCT116, were also used for the Western blot, as controls for the breast cancer cells. These were all cultured in RPMI 1640 medium supplemented with 10% fetal bovine serum (Corning, 35-011-CV), 100 UI/mL penicillin, and 100 mg/mL streptomycin (Corning, 30-002-CI), and 25 mmol HEPES. Cells were maintained at 37°C and 5% CO_2_. Cell lines were regularly passaged before the monolayer reached 80% confluency.

### Western blot

Western blot was used to measure FAAH expression in three breast cancer cell lines: MCF7, MDA-MB-231, and T47D. Along with the three breast cancer cell lines, three colorectal adenocarcinoma control cell lines (HT29, DLD1, and HCT116) were used for the second Western blot. All cells were cultured using the aforementioned culture protocol. Cell lysates were immobilized on a PVDF membrane and probed first with an anti-FAAH monoclonal antibody purchased from Abcam (ab54615), then stripped using Millipore Sigma’s ReBlot Plus Strong Antibody Stripping Solution and probed again for the loading control (α-tubulin or actin). Quantification was performed using Bio-Rad Image Lab™ software.

### Viability assays

Cells were plated in 96-well plates at a concentration of 5.0 × 10^4^ cells/well in 200 uL total media per well and allowed to acclimate overnight in an incubator at 37°C at 5% CO_2_. Cells were then treated with FAAH inhibitors (URB597 or PF-750), exogenous cannabinoids (AEA or PEA), or combination treatments of both FAAH inhibitors and exogenous cannabinoids (URB597 plus AEA or URB597 plus PEA) and placed back in the incubator overnight. Each plate corresponded to one of the treatments and included increasing dose concentrations for that specific treatment, as well as a negative control (cells and media), vehicle control (cells, media, and DMSO), and a blank (only media). Following the addition of treatments, the cells were again placed at 37°C at 5% CO_2_. Cell viability was measured using the MTT Cell Growth Assay Kit (CT02) from Millipore Sigma^®^ according to the manufacturer’s directions. Upon reduction of the yellow tetrazolium salt by NADH, a purple formazan crystal forms. Through this mechanism, MTT assays measure metabolic activity of cells; the darker the solution color in the wells, the greater the viability. Optical density readings were performed at 550 and 630 nm, based on the MTT Cell Growth Assay Kit instructions, using a BioTek^®^ ELx808 plate reader and analyzed with Gen5 software.

### Immuno-deficient mice

A cohort of 20 6-week-old female NU/J immune-deficient mice (also known as Athymic Nude, nu/nu) were obtained from Jackson Laboratories and housed in an SPF facility at the University of Houston Campus, following institutional animal review board approved protocol (Animal Protocol Number 16-008). Mice were allowed to grow to 8 weeks of age before being implanted subcutaneously with breast cancer tumor.

### Tumor implantation

Once MDA-MB-231 GFP-luciferase cells reached 70%+ confluency *in vitro*, they were removed as a single cell suspension using Trypsin-EDTA (0.25%) (Gibco, 25200-072). Density of cells was measured after staining with 0.4% Trypan Blue (Sigma, T8154) to exclude dead cells. To facilitate 3D growth, an injection vehicle of 50% Matrigel (Corning, 354234) and 50% PBS (HyClone, SH30028.02) was used. The Matrigel serves as a basement matrix and the PBS serves to dilute the solution for ease of injection and to avoid coagulation at room temperature. Cells were resuspended in 50/50 Matrigel/PBS at a concentration of 20 million cells per m. 100 uL of this solution was injected subcutaneously near the left mammary fat pad into each mouse.

### Animal treatment timeline

After injection, the breast cancer tumors were allowed to develop for one week. The mice were then imaged using the IVIS system and tumor burden was quantified. Since not all tumors were engrafted, the mice were then split into two groups of equal tumor burden using IVIS intensity data (*n* = 8 each). The control group received a vehicle treatment of 100 uL DMSO, while the experimental group were treated with 10 mg/kg URB 597 dissolved in DMSO. The mice were treated 3x/week and tumor burden was measured using calipers 3x/week. The experiment was run for a total of 10 weeks until we observed that the control group had reached terminal tumor burden, which was a tumor larger than 100 cm^2^, in accordance with the approved protocol. Mice were then euthanized using CO_2_ in combination with cervical dislocation to ensure mortality.

## SUPPLEMENTARY MATERIALS


