# Supplementary material for: FAAH inhibition ameliorates breast cancer in a murine model
Source: Oncotarget. 2023 Oct 31;14:910–8. doi: 10.18632/oncotarget.28534 (PMC10624203; doi:10.18632/oncotarget.28534)
Supplement: Supplementary file 1 [file oncotarget-14-28534-s001.pdf]

FAAH inhibition ameliorates breast cancer in a murine model

SUPPLEMENTARY MATERIALS

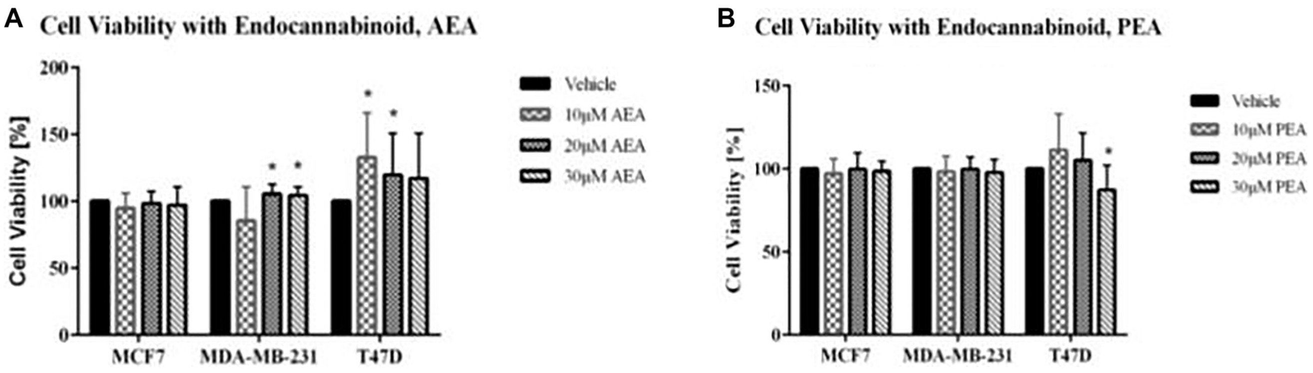

Supplementary Figure 1: Impact of exogenous cannabinoids on breast cancer viability. Cell viability assays were also conducted with exogenous cannabinoids, AEA (**A**) and PEA (**B**), using a vehicle control and AEA/PEA treatments at the following concentrations: 10 µM, 20 µM, and 30 µM. Statistical significance was determined using a non-parametric Mann-Whitney *U*-test ($^{*}p < 0.05$).
